# Carbohydrate flow during grain filling: Phytohormonal regulation and genetic control in rice (*Oryza sativa*)

**DOI:** 10.1111/jipb.13904

**Published:** 2025-04-07

**Authors:** Bohan Liu, Shuan Meng, Jianchang Yang, Jun Wu, Yan Peng, Jianhua Zhang, Nenghui Ye

**Affiliations:** ^1^ College of Agronomy Hunan Agricultural University Changsha 410128 China; ^2^ Yuelushan Laboratory Changsha 410128 China; ^3^ Key Laboratory of Crop Genetics and Physiology of Jiangsu Province Yangzhou University Yangzhou 225009 China; ^4^ Department of Biology Hong Kong Baptist University Hong Kong 999077 China; ^5^ School of Life Sciences and State Key Laboratory of Agrobiotechnology The Chinese University of Hong Kong Hong Kong 999077 China

**Keywords:** carbohydrate, environmental response, genetic control, grain development, grain filling, phytohormonal regulation, rice

## Abstract

Both the filling and development of grain are key processes determining agriculture production and reproductive growth in rice. The processes of grain filling and endosperm development are crucial for the accumulation of major storage compounds in rice grains. This requires extensive remobilization of carbon reserves from source to sink and the precise regulation of sucrose‐to‐starch conversion. Both the developmental sequence of the panicle and environmental signals influence the carbon flow between the leaves, leaf sheath, stem, and spikelets during grain filling. This, in turn, affects endosperm development and the production of storage compounds. In this review, we synthesize recent insight into grain development in rice, focusing on the dynamic changes in phytohormones and how their homeostasis integrates developmental and environmental cues to control grain filling in the developing panicle. We also highlight recent advances in the genetic control of carbohydrate remobilization and the transcriptional regulatory networks governing carbohydrate metabolism and grain development in rice. The asynchronous initiation and imbalance in grain filling limit the full yield potential of cereal crops. The “superior/inferior spikelets” serve as a model system for understanding the regulatory mechanisms underlying grain filling and development. Systematic research on carbohydrate flow and phytohormone crosstalk could enhance our understanding of optimizing yield production in cereal crops. Additionally, a thorough analysis of key genetic regulatory mechanisms can offer a genetic foundation and targets for precisely adjusting grain filling traits, ultimately aiding in the development of high‐yield crop varieties.

## Graphical abstract

This review examines how hormonal homeostasis integrates environmental/developmental signals to guide carbohydrate flow in rice grain filling and endosperm development. Genetic/transcriptional networks, explored through superior/inferior spikelet models, will help reveal targets to optimize grain filling and guide breeding high‐yield crops.

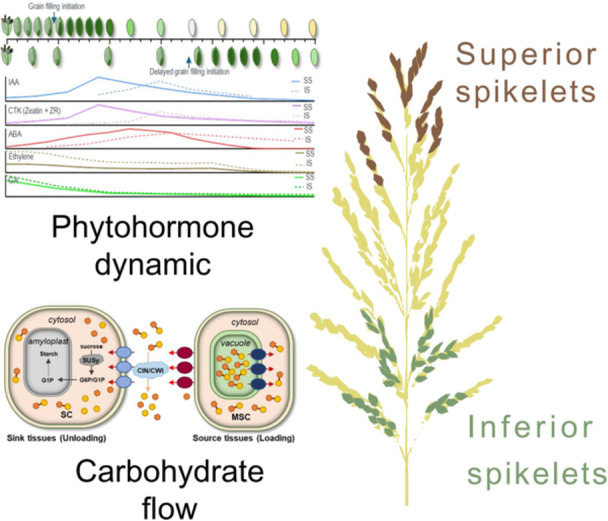

## INTRODUCTION

Rice (*Oryza sativa* L.) is a cereal crop belonging to monocots, as well as the most important staple food in the world (Muthayya et al., 2014). The rice grain consists of a diploid embryo, triploid endosperm and maternal tissues (testa and carpodermis) (An et al., 2020). The endosperm occupies most of the grain's space and serves as an important carbohydrate source for humans. During endosperm development, grain filling is primarily a heterotrophic process. At this stage, photosynthesis alone cannot meet the high demand for simultaneous grain filling, requiring the mobilization and reutilization of significant carbon reserves stored in maternal tissues (Chang and Zhu, 2017; Wang and Zhang, 2020). The translocation of carbohydrates from the source organ to the sink organ, and sugar metabolism and starch synthesis largely determine the yield and quality of rice. All of these processes required timely coordination between different source and sink organs by precise and complex regulatory mechanisms, which are mediated by genetic factors and phytohormones (Ma et al., 2023).

In paddy fields, the grain filling and development of rice grains are initiated asynchronously across the panicles (Ishimaru et al., 2003). The spikelets located on the upper primary rachis branches flower earlier (Superior spikelets (SS)), caryopses in these spikelets develop earlier and show the highest grain filling rate and the heaviest grain weight (Mohapatra et al., 1993; Patel and Mohapatra, 1996). In contrast, caryopses in later flowered lower secondary rachis branches spikelets (Inferior spikelets, IS) develop later and show the lowest grain filling rate and the lowest grain setting rate (Ishimaru et al., 2003; Panigrahi et al., 2023). Investigations regarding the grain filling difference between SS and IS demonstrated that the poor grain filling of IS was associated with insufficient enzyme activity in carbohydrate metabolism and altered sugar status (Ishimaru et al., 2005; Mohapatra et al., 2009). Conversely, phytohormone content such as auxin, cytokinin, and abscisic acids (ABA) in SS significantly differs from IS during the early grain filling stage (Yang et al., 2000, 2006b; Ishimaru et al., 2005; Zhang et al., 2009). Research using a superior spikelet/IS model allows the study of physiological factors that regulate grain filling and development.

Field studies have reported that moderate drought treatment during the mid to late stages of rice grain filling can affect plant hormone levels, significantly enhancing the grain filling rate of IS and improving their efficiency in converting and utilizing photosynthetic products (Yang et al., 2001, 2003; Yang and Zhang, 2006; Xu et al., 2007). Moreover, manipulation of phytohormone level on developing panicles also has a significant impact on the grain filling rate and grain yield in rice (Patel and Mohapatra, 1992; Yang et al., 2006b; Javid et al., 2011; Wang et al., 2015; Wu et al., 2016a). These studies emphasize the crucial role of phytohormones in regulating grain filling and ensuring proper developmental progression of the grain (Zhang et al., 2020). Phytohormones are critical endogenous substances that regulate plant physiological and developmental processes. Their levels fluctuate dramatically at specific time points during key events in grain development, highlighting the complex and unique roles each phytohormone plays in regulating rice grain filling and development (Basunia and Nonhebel, 2019; Wang and Zhang, 2020; Zhang et al., 2020). In developing spikelets, the dynamic changes in phytohormone levels are jointly regulated by the balance of phytohormone biosynthesis, metabolism, and transport. These processes, in turn, are influenced by both endogenous developmental signals and external environmental cues (Yang et al., 2001, 2003; Xu et al., 2007). In the superior/inferior spikelet model, the external application of phytohormones can alter the grain filling rate, final grain weight, and yield of both superior and IS. Therefore, studying how external factors and internal developmental signals regulate phytohormones, as well as identifying the key genetic factors that influence grain filling and development, can provide valuable genetic resources and theoretical guidance for improving cereal crops and developing high‐efficiency grain filling varieties. In this context, data from grain filling defect mutants, phytohormone‐related mutants, hormone measurements, external hormone applications, and transcriptomic studies contribute to developing a detailed model of phytohormonal regulation in grain filling and development.

Recently, molecular and genetic studies have identified key transcription factors that regulate starch synthesis, sugar transport, stress responses, and grain development in rice. These findings offer insight into the complex regulatory networks governing grain filling and endosperm development. Therefore, a systematic review is needed to evaluate recent advances in this research field.

In this review, we synthesized recent insight into the timeline of grain development in rice, offering a clear overview of storage compound accumulation, as well as anatomical changes and organ development. We reviewed how phytohormones change and their regulatory effect on grain filling and development. Next, the major pathways of sugar transportation and carbohydrate metabolism were reviewed as the most important biological processes during grain development and grain filling. Subsequently, we discussed the current progress in understanding how transcription factors respond to external and hormonal signals to regulate sugar metabolism, sugar transport, starch synthesis, and developmental processes. These molecular signaling networks ultimately influence grain filling and development in rice. This review will summarize our understanding of the phytohormonal and genetic regulation of grain filling and development, and provide an outline for further systemic studies for crop improvement and breeding.

## PHYTOHORMONAL DYNAMICS AND REGULATION IN RICE GRAIN DEVELOPMENT

### Grain development and maturation in rice

**Figure 1:**
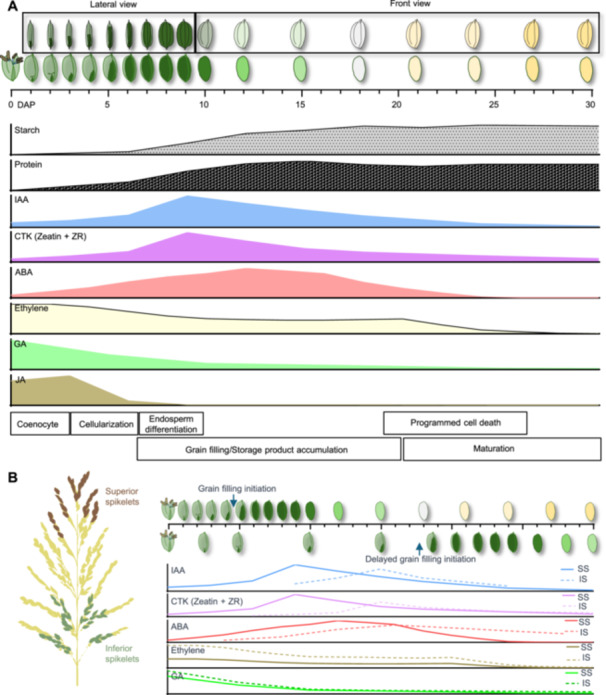
Schematic overview of grain development in rice **(A)** The relative volume of caryopsis within the grain and morphological event is presented throughout the developmental process of rice grain. Total starch and protein content in grain (relative to maximum volume) is shown in parallel with the time course (days after pollination (DAP)). **(B)** Timeline diagram of developmental differences and hormone level changes between superior and inferior grains. The solid lines represent superior spikelet grains (SS), and the dashed lines represent inferior spikelet grains (IS), illustrating the developmental differences and changes in hormone levels over time.

In rice, several studies have been carried out to provide a detailed description of this developmental process (Becraft and Yi, 2011; Wu et al., 2016b, 2016c). When rice is grown under a suitable night and day regime, grain development takes roughly 30 (±3) days (Wu et al., 2016b) (Figure 1A). The pollination event is referred to as DAP0 (day after pollination), the marker of this stage is the opening of the lemma and palea, revealing the stamens, which signifies flowering. The early morphogenesis will be initiated by the double fertilization of the embryo sac, as the coenocytic development stage (DAP 0–3) (Olsen, 2020) (Figure 1A). During this stage, the zygote (2n) development initiates with the fertilized oocyte, then divides asymmetrically and polarizes into basal and apical groups, apical parts are progenitors of the scutellum whereas the residual cells at the basal third form the embryo (Hoshikawa, 1993; Mohapatra and Sahu, 2022). From DAP4 to DAP12, the developing rice embryo undergoes key developmental stages, including the formation of the shoot apex, coleoptile, and leaf primordia, along with rapid growth and differentiation of the scutellum and vascular bundles (Hoshikawa, 1993) (Figure 1A). By DAP25, the embryo's morphological development is mainly complete, and it enters physiological dormancy as water content decreases, preparing the grain for harvest (Mohapatra and Sahu, 2022) (Figure 1A). The endosperm (3n) development begins with the fertilized central cell, through a series of programs, such as endoreduplication, and mitotic cell divisions, and acquires the basic plan of the endosperm coenocyte (Basunia and Nonhebel, 2019). The early morphogenesis development stage will finish within 1–2 DAP (Wu et al., 2016b). After this, the embryo and endosperm development continues, and the primary endosperm nucleus undergoes synchronous divisions for 3–5 days to accomplish the cellularization of endosperm, during this stage the rice caryopsis achieves major extension along the long axis (DAP 3–6) (Wu et al., 2016b). During this period, the caryopsis exhibits a green color and the highest water content (~80%) (Horigane et al., 2001). After this, the grain development enters the storage reserve accumulation stage (DAP 6–20). Between DAP 6 and DAP 9, endosperm cells continue to differentiate and expand, contributing mainly to the transverse growth of the grain during this period. With the continuing cell differentiation and accumulation of different storage products (DAP 10–18), the difference between the starchy endosperm (filled with starch grains and protein bodies) and aleurone layer (filled with lipid bodies and protein bodies) becomes more distinguishable (Figure 1A). Along with the starch accumulation in endosperm, programmed cell death (PCD) initiates from the central region of the starchy endosperm at DAP 8, and expands to the whole endosperm by DAP 18 (Wu et al., 2016c). Along with the storage product accumulation stage ending (DAP 6–20), the greenish grain will turn a pale yellow (Figure 1A). From DAP 21 to DAP 30, grain development enters the maturation stage, marked by starch crystallization and endosperm dehydration (Horigane et al., 2001; Wu et al., 2016c). At the end of the maturation stage, the grain husk will turn golden yellow and become fully matured.

### Phytohormones integrate both developmental and environmental signals to regulate grain filling and grain development

The phytohormones are the most important endogenous signals in plants, they are not only involved in the stress response and tolerance, but also participate in developmental programming such as aging and senescence. In particular, the biological processes of grain development and grain filling consist of several crucial events such as cell proliferation, differentiation, maturation and death. With rapid morphological changes within seeds, the level of phytohormones fluctuates differentially with the activities of metabolism, transportation and biosynthesis of genes (Zhang et al., 2020) (Figure 1A). Mutation or inhibition of phytohormone biosynthesis, transportation and metabolism in developing caryopsis results in grain filling defects and broad developmental defects of reproductive organs (Basunia and Nonhebel, 2019; Zhang et al., 2020). Application of exogenous phytohormone on the developing panicle also influences the stress response, storage compound accumulation and grain development in rice (Xu et al., 2007; Kim et al., 2009; Javid et al., 2011). Conversely, not only environmental cues such as drought and high temperature, but also developmental signals such as senescence induce changes in both phytohormone hemostasis and grain filling rate in developing panicles (Wang et al., 2015, 2019, 2020a; Cao et al., 2016). Thus, understanding the phytohormonal control mechanism in grain filling and development will provide us with significant insight into improving grain yield in rice.

#### Abscisic acid homeostasis is crucial for grain filling and endosperm development

Abscisic acid is a phytohormone associated with stress response, it is not only involved in triggering the seed maturation process, but also plays an important role in stress responses and tolerance (Phillips et al., 1997; Conrad and Fiedler, 1998; Sato et al., 2018). In the developing caryopsis, ABA begins to accumulate with the onset of endosperm cellularization at 3 DAP and continues to increase, reaching its peak at 15 DAP. This peak in ABA levels corresponds with the maximum grain filling rate (Wang et al., 2015; You et al., 2016; Zhang et al., 2020) (Figure 1A). In developing panicles, not only the ABA content but also the expression level of ABA biosynthesis genes *OsNCED1* and *OsNCED5* in IS was generally lower compared with SS (Tsukaguchi et al., 1999; Wang et al., 2015) (Figure 1B). Exogenous application of 20 μM ABA on the developing panicle significantly rescued the low rate of grain filling, cell division and starch biosynthesis in IS during the storage product accumulation stage (Grain filling stage), while application of the ABA biosynthesis inhibitor fluridone had the opposite effect on grain filling‐related processes (Yang et al., 2006b; Wang et al., 2015). These results demonstrate that ABA synthesis and accumulation are affected by the developmental sequence of the developing panicle, and the insufficient ABA content in IS is responsible for the grain filling defect. Conversely, field experiments showed that applying moderate drought stress during the mid to late grain filling stages in rice can significantly increase endogenous ABA levels, suppress the ABA catabolic gene *ABA8ox2*, and enhance grain filling in IS. However, severe drought stress led to overaccumulation of ABA, which inhibited grain filling, resulting in poor spikelet fertility and yield loss (Yang et al., 2001, 2006a, 2006b; Wang et al., 2019, 2020a). These phenomena indicate that ABA homeostasis responds to environmental signals and that the proper levels of ABA are crucial for grain filling in rice. Recently, a multidrug and toxic compound extrusion (MATE) transporter mutant *defective grain‐filling 1* (*dg1*), which was unable to translocate leaf‐derived ABA to the caryopsis, displayed an incompletely filled caryopsis and floury endosperm phenotype (Qin et al., 2021). These findings propose that the long‐distance translocation of leaf‐derived ABA is crucial for the proper maintenance of ABA homeostasis in sink organs, thus ensuring proper grain filling during rice grain development (Qin et al., 2021). Overall, ABA critically governs rice grain filling by spatiotemporally regulating endosperm cellularization, starch biosynthesis, and stress adaptation, with its homeostasis modulated by genetic factors (e.g., OsNCED1/5) and environmental signals (e.g., drought). Sustained ABA transport (via DG1) and optimal concentration thresholds ensure sink organ development, balancing yield formation under dynamic physiological and ecological constraints.

#### Dynamic regulation of auxin is associated with grain development

Auxins (indole 3‐acetic acid (IAA)) are plant hormones controlling plant growth and development, a substantial effort from recent studies has been made to understand its role in regulating grain filling and development (Basunia and Nonhebel, 2019; Zhang et al., 2020). In developing seeds, IAA levels begin to increase within 1 day after pollination and continue to rise during endosperm cellularization and aleurone differentiation, reaching their peak by the early stages of starch biosynthesis (DAP 4–DAP 10) (Uchiumi and Okamoto, 2010; Abu‐Zaitoon et al., 2012; Zhang et al., 2020) (Figure 1A). The concentration of active auxin in developing tissues results from the combined effects of auxin biosynthesis, transport, storage, activation, inactivation, response inhibition, and degradation. Therefore, the disruption of key genes involved in these processes can disturb the dynamic balance of IAA and lead to significant defects in grain development and filling (Zhang and Peer, 2017). Mutation of *DIOXYGENASE FOR AUXIN OXIDATION* (*DAO*) leads to defects in auxin oxidation and inactivation, causing excessive auxin accumulation in anthers and ovaries. This results in the production of parthenocarpic seeds without the need for fertilization (Zhao et al., 2013). Loss‐of‐function of IAA‐glucose hydrolase *THOUSAND‐GRAIN WEIGHT 6* (*TGW6*) in rice, causes insufficient IAA levels during the transition from the syncytial to the cellular stage, leading to an increase in endosperm cell layers and ultimately enhancing grain length and weight (Ishimaru et al., 2013). For IAA transport, a mutant named *big grain1‐D* (*Bg1‐D*) demonstrated that activation of *BG1* could strengthen auxin transport and resulted in larger grain (Liu et al., 2015). Disruption of *OsYUC11* in rice caused a cessation of IAA biosynthesis during the storage product accumulation phase, and the *osyuc11‐1* mutant exhibited a grain filling defect phenotype as chalky endosperm and weight‐reduced grain (Xu et al., 2021). For other IAA biosynthesis genes, like *OsYUC9* and *OsTAR1*, the knockout mutants of these genes also displayed similar grain filling defect phenotypes as *OsYUC11* (Xu et al., 2021). Moreover, endosperm‐specific expression of bacterial auxin‐synthesizing gene *Iaam* increased grain length and grain weight in rice (Zhang et al., 2020). These findings demonstrate that various metabolic pathways are involved in IAA homeostasis—such as oxidation, hydrolysis, transport, and biosynthesis—and play indispensable roles in key events of grain development and filling. In developing panicles, the IAA content in IS is significantly lower than in SS. The exogenous application of IAA to developing panicles has been shown to promote grain filling and increase grain weight in rice (Jin et al., 2002; Zhang et al., 2009). Conversely, abiotic stress significantly decreases the IAA content in developing panicles, and reduces the grain setting rate and grain weight in rice (Wu et al., 2016a, 2019). Overall, the dynamic and precise regulation of auxin levels is essential for the development of reproductive organs, as auxin plays an active role in regulating grain filling and endosperm development in rice. However, further investigation is required to clarify the downstream molecular mechanisms within the auxin signaling pathway that control grain filling and development.

#### Cytokinin function in regulating cell division of early endosperm development

Cytokinin was first report to positively regulate cell division by affecting the cell cycle, and play an important role in organ development (Miller, 1961; Jameson and Song, 2016). The zeatin and zeatin riboside content in developing rice seeds increases immediately after pollination and peaks around DAP 6 then declines until it became undetectable at DAP10 (Zhang et al., 2020). The cytokinin biosynthesis rate‐limiting *ISOPENTENYL TRANSFERASE* (*IPT*) genes *OsIPT4*, *OsIPT5*, and *OsIPT7* were also reported to be specifically expressed in endosperm and increased with the event of nuclear and cell proliferation (Zhang et al., 2020). Knockout of the *CYTOKININ OXIDASE/DEHYDROGENASE* (*CKX*) family gene *OsCKX2*, resulted in higher cytokinin content and larger seeds in rice (Yeh et al., 2015; Tsago et al., 2020). Conversely, *OsCKX11* is induced by ABA and leaf senescence and degrades cytokinins, particularly *trans*‐zeatin and *cis*‐zeatin (Zhang et al., 2021). In the *osckx11* mutant, elevated cytokinin levels resulted in increased branching, tillering, and grain number, while ABA levels decreased due to the downregulation of biosynthesis genes (Zhang et al., 2021). This demonstrates that *OsCKX11* coordinates the source–sink relationship in rice by regulating both cytokinin and ABA signaling, affecting leaf senescence and grain number (Zhang et al., 2021). A dominant mutant *big grain3* (*bg3‐D*), which overexpress *BG3/OsPUP4*, resulted in increased cytokinin content in the developing panicle and enlarged grain size in rice (Xiao et al., 2019). *OscZOG1* encodes a putative *zeatin o‐glucosyltransferase*, which mediates the reversible deactivation of zeatin; the knockdown of *OscZOG1* increased grain number and grain size in rice (Shang et al., 2016). Endosperm‐specific promoter‐driven *OsIPT3* expression demonstrated that elevated endogenous zeatin levels increased seed length and suppressed seed width (Zhang et al., 2020). Phytohormone analysis indicated that cytokinin levels in IS were generally lower than those in SS (Wang et al., 2006; Zhang et al., 2009) (Figure 1B). Moreover, external abiotic stress also decreased cytokinin content in developing panicles and decrease rice yield (Wu et al., 2016a, 2019). Conversely, several studies demonstrated exogenous cytokinin application could promote rice grain filling and carbohydrate accumulation (Javid et al., 2011; Panda et al., 2018). These results demonstrated that cytokinin orchestrated grain filling and seed development by spatiotemporal regulation of cell cycle progression and endosperm proliferation, mediated through biosynthesis genes (e.g., OsIPT4/5/7) and degradation pathways (e.g., OsCKX2/11). Their homeostasis, influenced by ABA antagonism and environmental cues, critically determines sink strength by modulating spikelet differentiation and carbohydrate allocation, with elevated cytokinin levels in SS or through exogenous application enhancing grain size and yield potential.

#### Ethylene is involved in the regulation of grain filling and leaf senescence

Ethylene is a gaseous hormone that plays an important role in plant stress responses and organ development. The ethylene evolution rate and ACC content initiated immediately after pollination, and reached the highest peak at DAP 2, then decreased with storage product accumulation (DAP 6–14), reached a plateau during DAP 15–20 and then continued to reduce with PCD to the lowest level (Yang et al., 2006b) (Figure 1A). In developing panicles, IS has a relatively higher ethylene evolution rate and ACC content compared with SS (Yang et al., 2006b) (Figure 1B). Application of either ethylene biosynthesis inhibitor Co(NO_3_)_2_ or ethylene antagonist 1‐methylcyclopropene (1‐MCP) promotes endosperm cell division, increases rice grain size and induces starch synthesis (Naik and Mohapatra, 2000; Panda et al., 2016). Conversely, ethephon (an ethylene releasing agent) treatment reduced cell division rate, grain filling rate, and grain weight in rice (Yang et al., 2006b). These studies demonstrated that excessive ethylene inhibits grain filling and a relatively lower level of ethylene is preferred during the rice grain filling stage. Ethylene has been regarded as a stress‐related hormone, abiotic stress induced the production of ACC and reduced grain weight and yield of rice (Hays et al., 2007). Suppression or knockout of ethylene receptor gene *ETR2* increased grain weight and delayed flowering in rice (Wuriyanghan et al., 2009). Mutation of another ethylene signaling central component gene, *MHZ7/OsEIN2*, reduced grain weight and delayed leaf senescence, whereas constitutive expression of *MHZ7/OsEIN2* promoted increased grain size and dark‐induced leaf senescence (Ma et al., 2013). These results indicated that ethylene acts as a negative regulator of grain filling in rice. Targeted suppression of ethylene signaling (via ETR2 inhibition) or biosynthesis (using 1‐MCP) enhanced sink strength and grain weight, highlighting the importance of maintaining low ethylene activity during grain maturation to mitigate stress‐induced yield penalties.

#### Gibberellins synthesized from embryos regulate endosperm development

Gibberellins (GAs) have essential signaling functions in key processes of plant growth and development, and their role in regulating seed germination has been widely researched in recent years. However, there have been only a few reports about gibberellins involved in grain filling and development. The content of GA_1_ and GA_4_ peaked at DAP3 and gradually decreased until DAP 30 (Zhang et al., 2009) (Figure 1A). Research on developing panicles demonstrated that IS has a relative higher GAs content compared with SS, and that a lower ABA‐to‐GA ratio can result in a low grain filling rate (Yang et al., 2001, 2003; Zhang et al., 2009) (Figure 1B). However, the exogenous application of GA_3_ to spikelets had no significant effect on total cell number and grain filling rate (Zhang et al., 2009). In contrast, overexpressing the GA biosynthesis gene GA20ox2 specifically in the endosperm increased grain length, suggesting that GA may play a role in regulating later stages of endosperm development (Zhang et al., 2020). In seeds, the GA biosynthesis genes were demonstrated to be specifically expressed in the embryos of germinating seeds as well as in developing grains in rice (Kaneko et al., 2003; Xue et al., 2012; Zhang et al., 2020). These results demonstrated that GA may be synthesized in embryos and transported to the endosperm and other tissues in developing seeds (Zhang et al., 2020). The phytohormone test demonstrated that GA content was relatively higher in inferior spikelets and lower in SS (Jin et al., 2002). In summary, gibberellins may influence grain filling and endosperm development by regulating the growth and physiological activities of the embryo, indirectly affecting embryo–endosperm interactions.

#### Role of other phytohormones in the regulation of grain filling and development

Other phytohormones, such as brassinosteroid (BR), jasmonate (JA), salicylic acid (SA), and strigolactone (SL), are novel phytohormone families in plants. Among them, BRs are plant steroid hormones that have been demonstrated to be involved in the regulation of grain size and endosperm development in rice (Morinaka et al., 2006). Knockout of BR signaling components *OsBZR1/2/3/4*, *OsBAK1*, and *OsPPKL1/2/3* decreases grain length, while knockout of BR signaling negative regulator *OsGSK1/2/3/4* results in longer grain (Yuan et al., 2017; Liu et al., 2021). However, endosperm‐specific overexpression of the BR biosynthesis gene *OsDWARF4* increases both grain size and grain in rice (Wu et al., 2008; Zhang et al., 2020). Moreover, exogenous 24‐epibrassinolid (EBR) application was demonstrated to increase rice yield and promote grain filling in IS, which indicated that BRs positively regulate sink size and grain filling in rice (Wu et al., 2008; Li et al., 2018; Zhang et al., 2020).

JAs are key cyclopentanone phytohormones for plant inducible defense responses. Recently these were also reported to be involved in the regulation of spikelets and grain development (Kim et al., 2009). In developing rice grain, the JA content was highest in the ovary and DAF3 and then rapidly declined at 6 DAF, indicating that JA may participate in the early development of rice grain (Zhang et al., 2020). The endogenous JA level was increased by overexpression of the Arabidopsis jasmonic acid carboxyl methyltransferase gene (*AtJMT*) gene or exogenous application of MeJA in rice and resulted in altered spikelets development and reduced grain setting rate (Kim et al., 2009). In Arabidopsis, JAs were demonstrated to be involved in seed size regulation (Hu et al., 2021). However, there have been very few reports about the involvement of JAs in the regulation of rice grain size and grain filling rate.

Salicylic acid is a well known phenolic compound that acts in plant defense; exogenous application of SA could significantly strengthen stress tolerance in plants (Hayat et al., 2010). Recent studies have found that SA200 (200 mg/L) treatment increased grain filling rate and grain weight of rice inferior spikelets, especially in the late grain filling stage (Chen et al., 2017).

Strigolactones are newly discovered phytohormones that have been reported to regulate plant architecture (Arite et al., 2009; Wang et al., 2020c). Recent studies on SL‐related *d* mutants, demonstrated that either SL biosynthesis or signaling defects resulted in reduce endosperm size, while exogenous application of GR24 (a synthetic SL) partially rescued the endosperm size of SL biosynthesis mutant *d10* (Yamada et al., 2019). This finding indicate that SL was involved in the regulation of rice endosperm development.

## CARBOHYDRATE METABOLISM AND REDISTRIBUTION DURING GRAIN FILLING AND GRAIN DEVELOPMENT

**Figure 2:**
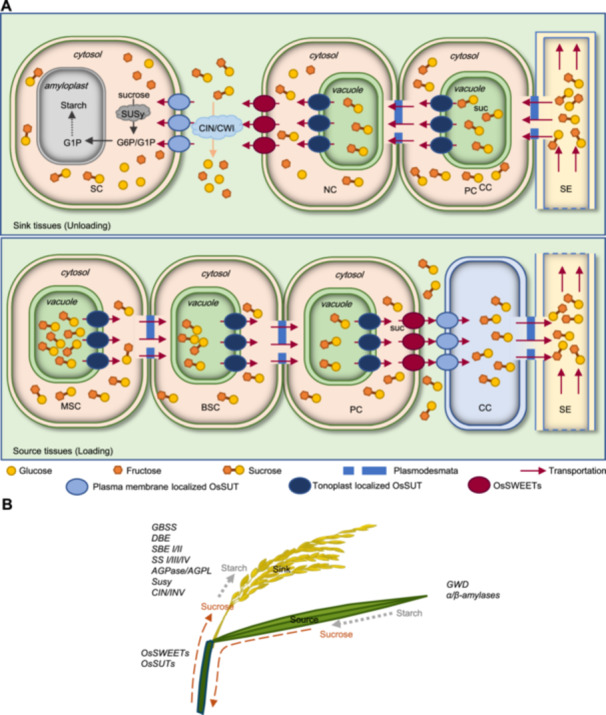
Schematic model of carbohydrate transportation and metabolism during grain filling and development **(A)** Possible transport routes of carbohydrates during rice grain filling. The model is made based on previous studies. In source tissues, sucrose may move from the mesophyll cell (MSC) to bundle sheath cell (BSC) and parenchyma cells (PC) in the leaf vascular bundle. The companion cell (CC) takes up sucrose and transfers sugar into the sieve elements (SE) in the phloem. In sink tissues, sucrose may unload from the phloem to PC in a vascular bundle and then transfer to nucellar cells (NC) through symplasmic transportation, then sucrose is exported into the apoplasmic space between nucellar cells and storage cells (SC). Clade III OsSWEET genes (*OsSWEET11/15*) involved in sucrose export into the apoplasmic space, and CIN/CWI gene (*GIF1*)‐mediated hydrolysis of sucrose are also important to the starch quality and grain filling rate. OsSWEET4‐mediated hexose transportation also plays an essential role in rice grain filling. **(B)** Schematic of carbohydrate transformation during grain filling and development. The dotted red lines indicate sucrose transportation routes, and the dotted gray lines represent the metabolic flow of carbohydrates. The major gene families involved in these bio‐processes are marked in the corresponding plots.

In rice (*Oryza sativa* L.), grain filling is actually a process of storage product accumulation along with rapid expansion of grain size, which is mainly a heterotrophic process. Grain filling is the key phase of rice reproductive growth, is closely associated with source organ senescence, pre‐stored assimilates remobilization, storage compounds synthesis and maturation (Lingan et al., 2015). The majority of storage products synthesized during grain filling are dependent on nutrients supplied by the maternal plant, including post‐anthesis photosynthetics and pre‐anthesis stored reserves. Carbohydrate supply for grain filling is mainly as sucrose from leaves and both starch and sucrose from leaf sheath and stems (Slewinski, 2012) (Figure 2A). An estimated 20%–40% of the grain mass consisted of previously stored reserves from source organs, which required proper senescence timing of source organs with nutrient recycling and remobilization (Scofield et al., 2007; Guiboileau et al., 2010).

During the grain filling stage, carbohydrates are sourced from starch degradation and sour sucrose synthesis in the source organ, then loaded into the phloem for remobilization to sink organs (Julius et al., 2017) (Figure 2A). Following a series of intracellular and extracellular reactions, the carbohydrates were converted to starch for long‐term storage in the amyloplasts of the sink organs (Huang et al., 2021) (Figure 2B). During these processes, sucrose is the predominant form of carbohydrate in long‐distance source‐to‐sink translocation (Liu et al., 2012) (Figure 2A). About 90% of mature rice grain consists of carbohydrates such as starch, therefore sucrose transportation and sugar–starch metabolism as important factors that impact the availability of carbohydrates, and play a crucial role in grain filling and organ development, ensuring a high and stable yield in rice (Kuanar et al., 2010).

### Sucrose loading from source tissues depends on SUT‐mediated intake activity

The sucrose uptake transporter (SUT) was first reported in spinach (*Spinacia oleracea* L.), where it mediated the active transport of sucrose and was involved in long‐distance transportation of sucrose (Riesmeier et al., 1992). In the rice genome, there are five genes that encode SUT proteins (Aoki et al., 2003). Among them, *OsSUT1*, *OsSUT3*, *OsSUT4*, and *OsSUT5* encode plasma membrane‐localized sucrose‐H^+^ cotransporters, while *OsSUT2* is localized in the tonoplast (Aoki et al., 2003; Eom et al., 2011). The tonoplast‐localized OsSUT2 is highly expressed in leaf mesophyll and bundle sheath cells but not in the vascular bundle (Eom et al., 2011) (Figure 2A). Mutation of *OsSUT2* results in more sucrose, fructose and glucose accumulation, and significantly reduced sugar export activity in *ossut2* mutant leaves (Eom et al., 2011). These results demonstrated that *OsSUT2* was responsible for vacuole‐to‐cytoplasm sugar transportation, thereby facilitating the movement of sucrose from source organs to other tissues.

Among plasma membrane‐localized OsSUTs, *OsSUT1* is the closest rice ortholog of *AtSUC2* and *ZmSUT1*, and is preferentially expressed in leaf phloem and stamens of mature spikelets, functioning as a sucrose importer (Hirose et al., 2010; Wang et al., 2021a) (Figure 2A). *OsSUT3* is highly expressed in developing pollen, *OsSUT4* is expressed in vascular bundles in the germinating embryo, in the aleurone layer of the caryopsis at the filling stage, while *OsSUT5* was found to be preferentially expressed in sheath phloem companion cells (Chung et al., 2014; Li et al., 2020; Wang et al., 2021a). The mRNA levels of *OsSUT1* and *OsSUT4* are much higher than *OsSUT3* and *OsSUT5* in mature and young leaves, leaf sheaths and roots, indicating that *OsSUT4* also plays an important role in sucrose transportation of rice aerial tissues (Wang et al., 2021a). Conversely, the pattern of preferential phloem companion cell expression in leaves implies that *OsSUT1/5* acts redundantly in apoplastic phloem loading (Wang et al., 2021a). In the model plant Arabidopsis, the single mutation of plasma membrane‐localized *AtSUC2* caused excessive carbohydrates accumulation in leaves and showed a retarded growth phenotype (Gottwald et al., 2000; Srivastava et al., 2008). However, CRISPR/Cas9 knockout of *OsSUT1/3/4/5* demonstrated that the single mutation of each plasma membrane‐localized OsSUTs had no significant impact on grain filling rate, but caused significant yield reduction in the plant (Wang et al., 2021a). These results indicated that plasma membrane‐localized *OsSUT1/3/4/5* play crucial roles in carbohydrate partitioning between source–sink organs during the grain filling stage. However, to clarify the detailed mechanism in plasma membrane‐localized OsSUTs and their role in phloem loading in rice, therefore impacting grain filling, a detailed analysis of double or triple mutants of OsSUTs will be needed.

### OsSWEET‐mediated sugar export activities are crucial for grain filling

The SWEET sugar efflux transporter family in plants was first identified by Förster resonance energy transfer (FRET) sensor‐based screening, and could be further divided into four clades (Eom et al., 2015). Among 21 rice SWEET genes, the Clade III SWEET proteins were demonstrated to be responsible for sucrose transportation, while Clades I and II are responsible for glucose transportation, and Clade IV for fructose transportation (Chen et al., 2012; Chen, 2014; Yuan et al., 2014). Most OsSWEET genes show higher expression levels in flowers and panicle branch tissues compared with other tissues, with the exception of OsSWEET12 and OsSWEET7a‐00 (Yuan et al., 2014). Among 21 SWEET genes in the rice genome, only *OsSWEET11/12/13/14/15* belong to the Clade III SWEET family and function as sucrose transporters (Chen et al., 2012).

By analysis and characterization of OsSWEET genes CRISPR/Cas9‐mediated knockout, demonstrated that mutation of Clade I/II OsSWEET genes (*OsSWEET1a*, *OsSWEET1b*, *OsSWEET4*, *OsSWEET6b*, *OsSWEET7c*) or Clade III OsSWEET genes (*OsSWEET11*, and *OsSWEET14*) could lead to both higher sugar accumulation but less starch content in mature seeds (Li et al., 2022a). These phenomena correlate the defects of sugar–starch conversion in developing grain with impaired SWEET‐mediated sugar transportation. Further investigation into the mutants of OsSWEET4, 11, 15 indicated that impaired transport of hexoses or sucrose led to severe grain filling defects, resulting in shrunken phenotypes in mature grains (Sosso et al., 2015; Ma et al., 2017; Yang et al., 2018). *OsSWEET4* is expressed in the base of the spikelet and plays a crucial role in exporting monosaccharides from maternal phloem to the developing caryopses (Sosso et al., 2015) (Figure 2A). *OsSWEET11* and *OsSWEET15* are expressed in the ovular vascular and the interface between the nucellar epidermis and the aleurone; the *ossweet11;15* double mutation causes excessive starch accumulation in the pericarp but less starch in the endosperm, and implies that these Clade III OsSWEET genes play a major role in apoplastic unloading of sucrose from the epidermis into developing caryopses (Yang et al., 2018) (Figure 2A). These data demonstrated that the OsSWEET‐mediated sugar export function is required to maintain proper apoplastic sugar transport in sink organs. Conversely, OsSWEETs have also been reported to play an important role in sugar efflux processes from source organs, and these processes are highly related to pathogen susceptibility (Chen, 2014; Wu et al., 2018). However, prospective data regarding *ossweet* multiple mutants and how their function in specific tissues in sink and source organs is needed to investigate OsSWEET‐mediated sugar transportation in grain filling and development.

### Sugar metabolism in source and sink organs is required for maintaining grain filling

Sugar metabolism processes such as starch degradation, sucrose synthesis in source organ, and sugar–starch conversion in sink organs are critical metabolism pathways to sustain the rice grain filling process (Jiang et al., 2021). In sink organs, the invertases and sucrose synthase (SuSy) are the two main enzymes that mediate the conversion of sucrose into glucose and fructose, and play an important role in promoting sucrose unloading and sink strength (Jiang et al., 2021) (Figure 2A). Conversely, ADP‐Glc pyrophosphorylases (AGPase), starch synthase (SS), starch branching enzyme (SBE) and starch debranching enzyme (DBE) play an important role in starch synthesis in sink organs.

Cell wall invertases (CWI/CIN) are immobilized enzymes located in the cell wall, and were demonstrated to promote sucrose unloading and starch synthesis in developing grain (Figure 2A). *OsGIF1/CIN2* encodes a cell wall invertase, and expresses ovular vascular and lateral stylar vascular traces during the storage product accumulation stage (Wang et al., 2008). Mutation of *OsGIF1/CIN2* causes severe weight reduction and more chalkiness in the *gif1* mutant grain, while overexpression of *OsGIF1/CIN2* increased grain production, indicating that cell wall invertase activity in vascular tissues is required for efficient starch synthesis during grain filling (Wang et al., 2008). Sucrose synthases are cytoplasmic enzymes that provide carbon for the synthesis of wall polysaccharides and starch (Chourey et al., 1998; Coleman et al., 2009; Jiang et al., 2012) (Figure 2A). Overexpression of *OsSUS* could significantly increase total grain yield and grain weight in rice, a detailed analysis of *OsSUS3* transgenic lines demonstrated that overexpression of *OsSUS3* promoted the grain filling rate and increased grain length, resulting in enhanced sink strength (Panda et al., 2015; Fan et al., 2019). The gain‐of‐function allele *WBR7* in sucrose synthase *SUS3* inhibits sucrose catabolism at conducting organs, redirecting carbon flux toward developing grains to boost storage compound biosynthesis, while reducing white belly rate (WBR) (Shi et al., 2024). Concurrently, elevated sucrose allocation to anthers fuels pollen viability and fertilization efficiency, synergistically enhancing both seed set percentage and yield potential (Shi et al., 2024). These results demonstrated that CIN and SUS activities are required for sucrose degradation and unloading in sink organs involved in the regulation of sink size and grain filling rates.

ADP‐glucose pyrophosphorylases (AGPase) control a rate‐limiting step in the starch biosynthetic pathway, mediate the conversion of glucose‐1‐P (G1P) and ATP to ADP‐glucose, and produce the major substrate for starch synthesis (Mohapatra et al., 2009). Mutation of either *OsGIF2/AGPL2* or *OsAGPS2*, which are the large subunit and small subunit of the cytosolic AGPase, respectively, decreases AGPase activity and results in decreased grain filling rate and shrunken endosperms with impaired starch synthesis in rice (Lee et al., 2007; Tuncel et al., 2014; Tang et al., 2016; Wei et al., 2017) (Figure 2B). Conversely, overexpression of OsAGPases in endosperm could increase seed size in rice (Tuncel and Okita, 2013). Other enzymes, such as *GBSSI/wx*, *DBE/ISA1*, *OsSSIIIa/Flo5*, and *BEIIb*, are involved in the conversion of ADPG to starch polymers amylose and amylopectin; mutation of these genes will significantly affect starch content and quality in rice grain (Tanaka et al., 2004; Kawagoe et al., 2005; Ryoo et al., 2007; Tian et al., 2009) (Figure 2B). In conclusion, starch biosynthesis is the final and most crucial biological process in sink organs, determining their strength as well as the final yield and quality of rice. Additionally, it influences the efficiency of carbohydrate flow within the plant.

In source organs, initiation of senescence, accompanied by degradation and translocation of preserved photosynthetic assimilates in leaf and leaf sheath, contributed 20%–40% of the total grain yield (Woo et al., 2019). *OsGWD1/LSE1* encodes an α‐glucan water dikinase which catalyzes the first step of starch degradation, while α‐amylase and β‐amylase play central roles in the degradation of starch to sugars (Hirose et al., 2013; Hirano et al., 2016; Li et al., 2021) (Figure 2B). Mutation of *OsGWD1/LSE1* led to excessive starch accumulation in leaves and a significant yield reduction, but overexpression of *OsGWD1/LSE1* increased final yield and increased grain length in rice (Wang et al., 2021b). Conversely, senescence‐specific expression of Ramy1A could promote starch degradation in leaf, stem and leaf sheath, and significantly promote grain filling rate in rice (Ouyang et al., 2021). Thus, increasing starch degradation in source organs could provide more carbohydrate translocation to sink organs, and promote grain filling and the final yield in rice. However, the detailed molecular mechanisms by which senescence signals in rice source organs induce starch degradation and promote non‐structural carbohydrate (NSC) transport remain to be further elucidated.

## TRANSCRIPTIONAL REGULATION OF CARBON ALLOCATION IN SOURCE–SINK COORDINATION DURING GRAIN FILLING

The coordinated “source–sink‐translocation” system governs grain filling efficiency in crop yield formation. Source organs, primarily functional leaves and senescing leaf sheaths, supply carbohydrates and nutrients through photosynthetic assimilation and remobilization. Leaves, as central source organs, not only drive photosynthetic carbon fixation but also regulate assimilate partitioning via senescence dynamics, critically impacting grain filling. Recent studies have revealed that this regulatory network integrates multilayered controls by sugar signaling, phytohormones, and senescence‐associated genes, with transcription factors serving as central regulatory hubs.

The translocation system, bridging source–sink interactions, encompasses both structural components (e.g., vascular bundles) and dynamic assimilate transport efficiency. Crop yield strategies are classified into three paradigms: source limited (inadequate assimilate supply), sink limited (restricted storage capacity), and source–sink coordinated (synergistic enhancement). Notably, sink organs bidirectionally regulate source photosynthetic activity and translocation efficiency through metabolic feedback, a process orchestrated by spatiotemporal expression of specialized transcriptional modules.

Sink organs, as the ultimate destination of carbohydrate flux, undergo distinct metabolic reprogramming during grain filling. Phloem‐unloaded sucrose is converted via an enzymatic cascade to glucose‐1‐phosphate (G1P) for starch biosynthesis. This transformation requires precise coordination of sugar transporters and dynamic rewiring of metabolic pathways, underpinned by spatiotemporally defined transcriptional networks that balance carbon allocation and storage capacity.

### OsNAC16 coordinates ABA‐driven senescence and source‐to‐sink nutrient allocation for grain filling in rice

**Figure 3:**
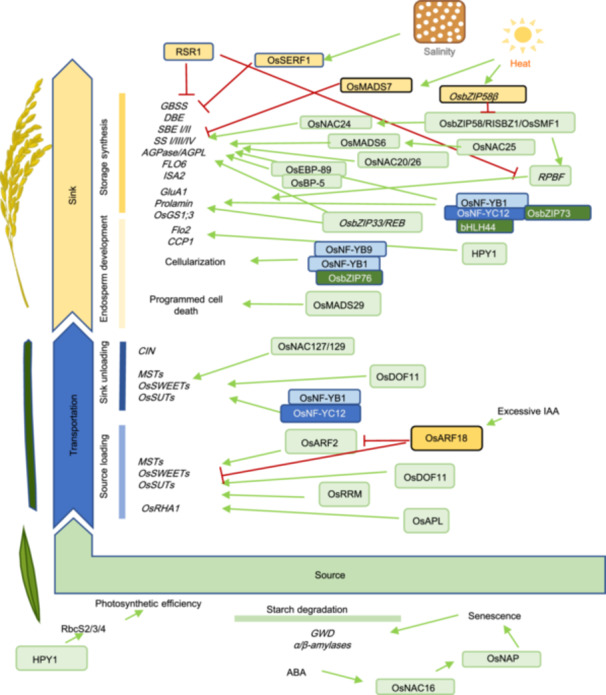
Schematic presentation of the regulatory network regulating grain filling, grain development and carbohydrate partitioning in rice Transcription factors and their downstream pathways are involved in starch degradation, carbohydrate transportation, storage synthesis and grain development in rice. Green rounded squares and yellow rounded squares represent positive regulators and negative regulators, respectively. Red blocking arrows and green arrows indicate inhibition and promotion, respectively.

Plant senescence is associated with the accumulation of ABA, which initiates the remobilization of carbohydrates in source organs. Recent research has demonstrated that OsNAC16 plays a pivotal role in regulating this process. Yeast‐one‐hybrid assays and dual‐luciferase reporter experiments have verified that OsNAC16 directly binds to the promoter of the *OsNAP* gene, thereby inducing its expression (Gi et al., 2024) (Figure 3). Moreover, *OsNAC16* is activated by ABA signaling, which promotes chlorophyll degradation, senescence, and the expression of genes within the ABA signaling pathway. These alterations facilitate the transfer of nutrients from source to sink, ultimately contributing to an increase in rice grain yield.

### OsNAC023 enhances rice yield by optimizing source–sink carbon flow through the Tre6P signaling pathway

Efficient sugar transport and conversion are essential for source–sink interactions. Recent studies have identified Tre6P as a sugar signal involved in regulating these interactions. In rice, *OsNAC023* has been found to mediate sugar signaling and the saturation status between source and sink organs by regulating trehalose metabolism (Li et al., 2022a, 2022b). OsNAC023 directly binds to the promoter of *OsTPP1*, repressing its expression and consequently promoting the accumulation of Tre6P (Figure 3). The elevated Tre6P enhances the photosynthetic rates in source organs, facilitates carbon transport to sink organs, and supports the development of panicles and seeds. Collectively, these effects lead to a substantial increase in the grain yield per plant.

### HPY1: Dual transcriptional control of leaf photosynthesis and grain filling in rice

Recently, several transcription factors with dual functions in regulating both source and sink have been identified. One such factor, HPY1, encodes a transposon‐derived protein containing HTH and DDE‐4 domains (Fan et al., 2023). As a transcription factor, HPY1 directly binds to the promoters of source‐related genes (*RbcS2*, *RbcS3*, and *RbcS4*, which encode RuBisCO small subunits), enhancing their transcription (Figure 3). This leads to an increase in RuBisCO content and activity, improving photosynthetic efficiency and biomass production. Simultaneously, HPY1 binds to the promoters of sink‐related genes (*CCP1* and *FLO2*, which regulate grain size and starch content), upregulating their expression and promoting grain development (Figure 3). Cloning such transcription factors can offer valuable insight into the genetic and molecular mechanisms underlying source–sink coordination in rice.

### OsAPL promotes rice grain filling by regulating nutrient transport from source to sink

Sucrose loading in source organs is crucial for the efficient transport of carbohydrates between source and sink organs. In source organs, a MYB‐type transcription factor, ALTERED PHLOEM DEVELOPMENT (OsAPL), has been identified, with specific expression in mesophyll and companion cells (Yan et al., 2022). OsAPL directly binds to the promoter of the plasma membrane H⁺‐ATPase gene *OsRHA1*, activating its expression (Figure 3). This promotes the transport of nutrients from source organs to sink organs, ultimately enhancing grain filling and increasing rice yield.

### OsDOF11 regulates sucrose export from source organs via OsSUTs and OsSWEETs

In rice, a transcription factor encoding DNA BINDING WITH ONE FINGER11 (OsDOF11), was found to be preferentially expressed in parenchyma cells and phloem companion cells in leaves and stems, as well as dorsal vascular bundles of developing seeds (Wu et al., 2018). Loss function of *OsDOF11* results in semi‐dwarf plants, fewer branched panicles and the production of fewer and smaller grains (Wu et al., 2018). Radioactively labeled [^14^C]sucrose uptake assays showed that sucrose uptake ability was significantly abolished in the *osdof11* mutant (Wu et al., 2018). Moreover, transcript levels of *SWEET* and *SUT* genes in young panicles, mature leaves and germinating seeds were significantly reduced in the *osdof11* mutant (Wu et al., 2018). Chromatin immunoprecipitation (ChIP) assays demonstrated that OsDOF11 could directly bind to the promoters of *OsSUT1*, *OsSWEET11*, and *OsSWEET14*, suggesting that OsDOF11 modulates sugar transportation by targeting these sugar transporter genes (Wu et al., 2018) (Figure 3). In addition, *OsDOF11* and *OsSWEET14* were demonstrated to be involved in resistance against *Rhizoctonia solani*, emphasizing the important role that OsDOF11 plays in apoplastic sucrose transportation (Wu et al., 2018; Kim et al., 2021).

### The nuclear factor Y family genes: Bridging sucrose allocation, starch biosynthesis and endosperm cellularization in sink organs

Nuclear factor Y (NF‐Y) family member gene *OsNF‐YB1* was first identified from aleurone‐specific genes by microarray analyses (Bai et al., 2016). The CRISPR‐knockout mutant *nfyb1‐1* and RNAi transgenic plant displayed a chalky endosperm phenotype, which indicated that the *OsNF‐YB1* mutation or knockdown caused a grain filling defect in developing caryopses (Bai et al., 2016). Further analysis showed that not only sucrose, fructose, and glucose contents, but also *SUT* gene expression, were significantly reduced in developing caryopses of the *nfyb1‐1* mutant, indicating that the sucrose uptake by rice endosperm was weakened by *OsNF‐YB1* mutation (Bai et al., 2016). Molecular analyses found that OsNF‐YB1 could bind to the promoter and activate the expression of *OsSUT1*, *OsSUT3* and *OsSUT4* (Bai et al., 2016). Another NF‐Y family member, OsNF‐YC12, was reported to interact with OsNF‐YB1 to regulate the expression of *OsSUT1* (Xiong et al., 2019) (Figure 3). Molecular analyses proved that *OsNF‐YC12* not only regulated starch synthesis by targeting *FLO6 (FLOURY ENDOSPERM6*) but also affected amino acid metabolism by targeting *OsGS1;3* (*glutamine synthetase1*) in rice developing endosperm (Xiong et al., 2019). Moreover, NF‐YB1 and NF‐YC12 were found to interact with bHLH144, therefore assembling into a heterotrimer complex, which activates granule‐bound starch synthase gene *Wx* to modulate grain quality in rice (Bello et al., 2019) (Figure 3). In another study, OsbZIP76 interacted with OsNF‐YB9 and OsNF‐YB1 regulated endosperm cellularization (Niu et al., 2020). In addition, one study reported that the transcription factor OsMYB73 was highly expressed in rice grains during the early stage of grain filling (Liu et al., 2024) (Figure 3). It was preliminarily elucidated that OsMYB73 collaborates with the nuclear factor OsNF‐YB1 (Liu et al., 2024). Together, they bind to genes such as the isoamylase gene *OsISA2*, the lipid‐transfer protein gene *OsLTPL36*, and the flavin‐monooxygenase gene *OsYUC11* (Liu et al., 2024) (Figure 3). In summary, the transcription factors encoded by NF‐Y family genes expressed in seeds play a central role in regulating key physiological processes such as sucrose uptake, starch synthesis, and amino acid metabolism, which are essential for caryopsis development. Reports on the interactions between NF‐Y genes and other transcription factors also highlight their pivotal position within the regulatory network.

### Seed‐specific NAC TFs orchestrate sucrose transport and starch biosynthesis for optimal grain filling

In the rice genome, there are 151 genes encoding NAC transcription factors, nine of them are reported to be seed specifically expressed NAC transcription factors (Mathew et al., 2016). Among them, OsNAC020 and OsNAC026 are expressed in the aleurone layer, starchy endosperm, and the embryo of rice grains, and are required to transactivate the expression of starch synthesis gene*s AGPS2b, AGPL2*, *SBEI*, *SSI*, and *Pul*, as well as protein synthesis genes such as *GluA1*, *glutelin B4/5*, *α‐globulin*, and *16 kD prolamin* (Wang et al., 2020b). The double mutation of *OsNAC020* and *OsNAC026* decreased starch and storage protein content, causing floury grain (Wang et al., 2020b) (Figure 3). OsNAC25 enhances OsNAC020/26 activity to amplify starch biosynthesis, while its overexpression forms non‐functional OsNAC25–OsNAC020/26 complexes, preventing promoter binding and avoiding transcriptional overload (Wang et al., 2024) (Figure 3). The OsNAC24–OsNAP complex binds novel motifs (TTGACAA/ACAAGA/ACAAGA/CACG) to activate key starch enzyme genes (e.g., *OsGBSSI*, *OsSBEI*), fine‐tuning amylose/amylopectin ratios and starch cooking properties (Jin et al., 2023). *ONAC127* and *ONAC129*, dominantly expressed in the pericarp during early and middle grain development stages, directly bind the promoters of and regulate the expression of sugar transporters *OsMST6* and *OsSWEET4* and heat stress response genes *OsMSR2* and *OsEATB* (Ren et al., 2021) (Figure 3). Additionally, *OsNAC023* and *OsNAC025* have also been reported to be associated with the regulation of seed size and starch synthesis (Mathew et al., 2016, 2020). Overall, these findings reveal that the NAC family achieves precise regulation of starch synthesis and resource allocation during grain development through spatiotemporally specific functional modules (endosperm core synthesis, testa nutrient transport) and self‐balancing networks (positive/negative feedback loops).

### OsbZIP58/RISBZ1/OsSMF1 couples source organ metabolic homeostasis with environmental sensing to ensure robust grain filling

*OsbZIP58/RISBZ1/OsSMF1* encoding close homologs of maize storage proteins master regulator *OPAQUE2* (*O2*) in rice, were reported to regulate starch and storage protein synthesis with *RICE PROLAMIN BOX BINDING FACTOR* (*RPBF*) (Kawakatsu et al., 2009). OsbZIP58 could bind to promoters of and regulate the expression of storage compound synthesis genes such as *OsAGPL3*, *Wx*, *OsSSIIa*, *SBE1*, *OsBEIIb*, *α‐globulin* and *ISA2* (Wang et al., 2013; Kim et al., 2017) (Figure 3). Moreover, OsbZIP58 also transactivates the expression of *RPBF* and *ONAC024*, indicating that OsbZIP58 plays an important role in the transcriptional regulation of grain filling (Kim et al., 2017) (Figure 3). In another study, *OsbZIP58* activity was demonstrated to be crucial for storage material accumulation under high‐temperature stress (Xu et al., 2020). High temperature induces alternative splicing of *OsbZIP58* into its truncated isoform *OsbZIP58β*, a lower transactivation activity variant (Xu et al., 2020) (Figure 3). Conversely, rice varieties that are more tolerant to high temperatures also have reduced alternative splicing of *OsbZIP58*, indicating that *OsbZIP58* positively regulates grain filling and high‐temperature tolerance (Xu et al., 2020).

### Auxin signaling cascade OsARF18–OsARF2 regulates carbohydrate partitioning and grain development

*Dioxygenase for Auxin Oxidation* (*DAO*) mediates the deactivation of auxin, and plays an important role in the homeostasis of auxin in developing anthers and ovaries (Zhao et al., 2013). In the *dao* mutant, excessive auxin accumulation induces *OsARF18* transcription levels and represses the expression of *OsARF2*. OsARF18 directly binds to the AuxRE motif of the *OsARF2* promoter and the SuRE motif of the *OsSUT1* promoter and inhibits the expression of *OsARF2* and *OsSUT1* (Zhao et al., 2013, 2022) (Figure 3). As a result, insufficient sugar supply in reproductive organs and sucrose accumulation in source organs was observed in the *dao* mutant (Zhao et al., 2022). In wild‐type plants under relatively lower IAA conditions, OsARF2 could activate its own expression, bind to the *OsSUT1* promoter, activate *OsSUT1* expression, and maintain proper carbohydrate partitioning and grain development (Zhao et al., 2022) (Figure 3). The OsARF18–OsARF2 cascade demonstrated the crucial role that auxin deactivation and auxin signaling played in the regulation of carbohydrate partitioning and grain development.

### Involvement of other transcription factors in the regulation of grain filling and maturation

*SALT‐RESPONSIVE ERF1* (*SERF1*) encodes the DEHYDRATION‐RESPONSE ELEMENT‐BINDING (DREB)‐type transcription factor, involved in salt stress tolerance (Schmidt et al., 2013). SERF1 directly binds to the promotors of *GBSSI and RPBF*, and negatively regulates grain filling and germination in rice (Schmidt et al., 2014) (Figure 3). *Rice Starch Regulator1* (*RSR1*), an APETALA2/ethylene‐responsive element‐binding protein family transcription factor, was identified by coexpression analysis to be negatively correlated with the expression of starch synthesis genes (Fu and Xue, 2010) (Figure 3). Another AP2/EREBP transcription factor OsEBP‐89 expressed in the developing endosperm and intercalary meristem, interacts with OsBP‐5, binds to the promoter of the *Wx* gene, and induces its expression (Yang et al., 2002; Zhu et al., 2003) (Figure 3). A rice endosperm‐specific bZIP TF, named *OsbZIP33/REB*, interacts with the promoters of *Wx*, *SBE1*, and *α‐globulin* genes in rice, and promotes starch synthesis and protein synthesis (Cai et al., 2002) (Figure 3). OsRRM, an RNA binding protein binds directly to and stabilizes the mRNA of sugar transporter genes, and is involved in the regulation of sugar transportation and signaling (Liu et al., 2020) (Figure 3).

## CONCLUDING REMARKS AND FUTURE PERSPECTIVES

### Decoding phytohormone interplay: Drivers and responders in the rice grain filling regulatory network

With developmental events occurring during rice grain filling and development, the content of various phytohormones fluctuates differentially with the activities of metabolism, transportation and biosynthesis genes. Furthermore, as intrinsic signal molecules are involved in the regulation of crucial developmental decisions, the content of phytohormones was also influenced by the external environment. The current understanding of phytohormone content during rice grain development provides us with a glimpse of each phytohormone effect. The increase in IAA/CTK content is accompanied by vigorous cell proliferation and differentiation. The ABA content reaches the highest peak and ETH content drastically decreases with massive storage product accumulation in rice grains (Figure 1A). Lower IAA/CTK/ABA and higher ETH contents in IS were demonstrated to be related to weaker grain filling (Figure 1B). Conversely, recent field tests have demonstrated that moderate drought treatment could increase ABA and IAA contents in IS and promote grain filling (Wang et al., 2015; Teng et al., 2022). Excessive nitrogen decreases IAA/CTK content and grain filling in IS (Chen et al., 2022). Saline–alkali stress inhibits grain filling and promotes ethylene production (Peng et al., 2022). The correlation between insufficient IAA, CTK and ABA contents or excessive ETH content and lower grain filling rates in IS, has not only been reported in rice but also in other cereals (Yang et al., 2006a; Cai et al., 2014; Du et al., 2021). In wheat and maize, although the traits included plant morphology, and seed morphology differed in rice, the phytohormonal effect on seed filling and yield production was similar to that of rice. In addition, evidence from recent research emphasized that other phytohormones such as GA, BR, JA, SA, and SL also participated in the regulation of grain development and grain filling (Yang et al., 2021). Emerging evidence has highlighted that exogenous hormone application induces reciprocal fluctuations among phytohormones, due to a crosstalk mechanism critical for coordinating source–translocation–sink dynamics. For instance, OsCKX11‐mediated cytokinin (CTK)/ABA antagonism coordinates leaf senescence and sink strength during grain filling (Zhang et al., 2021). Moreover, exogenous auxin (IAA) application suppresses ABA accumulation in developing grains, while reciprocal ABA–IAA antagonism modulates assimilate partitioning (Xu et al., 2007; Teng et al., 2022). Genetic studies have demonstrated that the downstream components of the hormone signaling cascade directly influence carbohydrate allocation, e.g., IAA signaling via auxin response factors OsARF2/OsARF18 regulates sucrose transporter OsSUT1 expression (Zhao et al., 2022) (Figure 3). These findings collectively establish phytohormone interplay as a pivotal mechanism through which grains integrate environmental, nutritional, and abiotic stress signals, while simultaneously driving transcriptional reprogramming to fine tune source–sink metabolic activities.

Overall, the homeostasis of phytohormone highly influences grain filling and endosperm development, excessive or insufficient phytohormone supply in developing gran will cause adverse effects on grain filling. However, most studies have focused on the physiological role that the phytohormones played in grain filling and development, so the upstream or downstream signaling and target pathways have largely remained elusive. Moreover, the translocation of phytohormones from maternal tissue during grain filling is an important source for phytohormone signaling in developing grain. This raises the question of how other parts of plants interact with the developing grain through phytohormone communication, and how phytohormones crosstalk with other hormones to regulate grain filling and carbohydrate partitioning. There is a need to use systematic approaches to dissect the complex regulatory networks behind these processes.

### Genetic control of rice grain filling: Challenges and solutions

The genetic regulation of grain filling represents a pivotal yet underexplored frontier in cereal crop improvement. While substantial progress has been made in characterizing key genes (e.g., *OsSUT*, *OsSWEET*) and transcriptional regulators (e.g., *OsDOF11*, *OsNF‐YB1*), several critical challenges persist, limiting our ability to fully harness these genetic insights for breeding applications.

Grain storage reserves and filling capacity predominantly depend on cumulative nutrient assimilation during the rice life cycle, demonstrating coordinated physiological interactions between source and sink organs (Figure 2). While achieving robust grain filling represents a central breeding objective, asynchronous filling patterns in cereal crops continue to hinder full yield potential realization. Recent studies have suggested that modulating phytohormone spatiotemporal distribution could optimize the grain filling processes: for example, *OsCKX11* knockout lines have demonstrated delayed leaf senescence through cytokinin regulation (Zhang et al., 2021), while targeted *OsABA8ox2* knockout enhances grain filling rates via ABA metabolism modulation (Teng et al., 2022).

Carbohydrates biosynthesized in source organs are converted into sucrose, subsequently undergoing long‐distance translocation to sink organs for starch biosynthesis. The OsSUT and OsSWEET gene families play indispensable roles in phloem loading and carbohydrate partitioning during grain filling (Figure 2). Targeted mutagenesis studies have revealed that coordinated hexose and sucrose provisioning through these transporters is essential for normal starch deposition in developing grains. However, a critical constraint persists in source–sink translocation efficiency. Current research suggests three potential strategies to address this limitation: (i) enhancing sucrose export by engineering transcriptional activators of sucrose transporters, such as OsDOF11 (Kim et al., 2021); (ii) increasing vascular transport capacity through modifying phloem development regulators like OsAPL (Yan et al., 2022); and (iii) reprogramming the OsNAC23–Tre6P signaling axis to optimize source‐to‐sink carbon allocation (Li et al., 2022b).

Finally, the efficiency of carbohydrate conversion, grain filling, and starch biosynthesis in sink organs is pivotal for achieving both high yield and superior grain quality. While recent studies have elucidated key genetic regulators of starch synthesis and grain quality in rice (e.g., *OsGBSSI*, *OsSSIIa*), emerging evidence highlights the detrimental effects of abiotic stresses—such as high temperature and drought—on these processes. Stress conditions disrupt sucrose partitioning, suppress starch synthase activity, and impair cellularization of the endosperm, ultimately reducing sink strength and grain weight (Zhang et al., 2017; Xu et al., 2020).

The third critical challenge lies in resolving the trade‐offs between sink strength and stress tolerance. Recent advances have identified stress‐responsive transcription factors (OsSERF1, OsMADS7, OsbZIP58) that modulate grain filling under salt or heat stress. To address this, strategic engineering of regulatory networks could stabilize sink activity under stress: (i) editing promoter regions of starch‐related genes (e.g., *SBE1*) to decouple their expression from stress‐responsive repressors like OsMADS7; and (ii) knockout of stress‐responsive repressor such as OsSERF1.

Although there are numerous examples of links between transcription factors, sugar transportation, starch synthesis and stress responses, there is little knowledge about the crosstalk between transcription factors and phytohormone signaling, as well as the endosperm–embryo communication. Thus, substantial effort in understanding the genetic control of grain filling and development will provide more genetic resources and a theoretical basis for cereal crop improvement and breeding.

## CONFLICTS OF INTEREST

The authors declare no conflicts of interest.

## AUTHOR CONTRIBUTIONS

B.H.L., J.H.Z., and N.H.Y. conceived the manuscript; B.H.L., S.M., and N.H.Y. drafted the manuscript; B.H.L, J.W., Y.P., J.C.Y, J.H.Z., and N.H.Y. revised the manuscript. All authors read and approved of the manuscript.
